# Probiotic effects on ectoparasitic mite infestations in honey bees (*Apis mellifera*) are modulated by environmental conditions and route of administration

**DOI:** 10.1128/spectrum.02498-24

**Published:** 2025-05-22

**Authors:** Andrew P. Pitek, Brendan A. Daisley, John A. Chmiel, Anna M. Chernyshova, Gurpreet Dhami, Gregor Reid, Graham J. Thompson

**Affiliations:** 1 Department of Biology, The University of Western Ontario 98604, London, Ontario, Canada; 2 Department of Molecular and Cellular Biology, University of Guelph, Guelph, Ontario, Canada; 3 Centre for Human Microbiome and Probiotic Research, Lawson Health Research Institute 151158 , London, Ontario, Canada; 4 Department of Microbiology & Immunology, The University of Western Ontario 468153, London, Ontario, Canada; 5 Department of Surgery, The University of Western Ontario 6221, London, Ontario, Canada; University of the Philippines Los Baños, Los Baños, Laguna, Philippines

**Keywords:** apiculture, managed pollinators, microbial therapeutics, microbiome, *Varroa* mite, American foulbrood

## Abstract

Beneficial bacteria can improve the health of managed honey bees (*Apis mellifera*) via immune system support as well as direct inhibition of pathogens. However, our understanding of how environmental factors and delivery methods impact treatment outcomes is limited. Here, we evaluated how supplementation of a three-strain lactobacilli consortium (*Lactiplantibacillus plantarum* Lp39, *Apilactobacillus kunkeei* BR-1, and *Lacticaseibacillus rhamnosus*; LX3) affects ectoparasitic mite (*Varroa destructor*) and bacterial (*Paenibacillus larvae*) load. We measured this effect following delivery of LX3 in either edible (protein patty) or topical (spray) forms and did so across three distinct habitats (forage-rich, agricultural, and urban). Results demonstrate significant control of mite infestation levels following patty delivery, relative to untreated patty controls. Spray methods, by contrast, were ineffective. Specifically, a control group that received only an uninoculated patty carried more mites than any other group, suggesting excess protein within hives is a catalyst for mite proliferation. This effect, whereby the excess-protein group had the highest parasite load, was pronounced in the most natural (forage-rich) environment type, indicating a significant site-by-treatment interaction. No influence of LX3 on *P. larvae* loads was observed in the asymptomatic colonies studied, which is likely attributable to the already low levels of pathogenic spores present and challenges associated with detection limits. In summary, this multi-site field study suggests that an LX3-infusion of standard protein patties is an effective method to control *Varroa destructor* infestations in commercial honey bee colonies.

**IMPORTANCE:**

Commercial beekeeping operations typically have a narrow profit range that depends on maintaining healthy hives throughout the season. Unfortunately, parasitic *Varroa* mites and bacterial pathogens can contribute to colony losses. The plight of honey bees can in turn affect the price and availability of produce on the agri-food market. There is therefore a need for innovation in the beekeeping industry to help secure bee livestock from season to season. One relatively new approach to sustainable beekeeping is the use of beneficial bacterial supplements that beekeepers can feed to or otherwise apply to hives in aid of the bee’s natural health and abilities as mediated through their gut-brain axis. Our multi-site field study applies this approach. We find that a pollen protein patty is an effective vehicle for delivering probiotic bacteria to commercial honey bee colonies and for helping to keep *Varroa* destructor infestation levels in check.

## INTRODUCTION

The Western honey bee (*Apis mellifera*) is an economically valuable insect whose natural ability to forage for pollen and nectar has rendered it an efficient pollinator of flowering crops (1). The strategic use of honey bees in the pollination services sector is thought to contribute to the production of as much as 35% of the world’s produce (2). *Apis mellifera* has, however, become increasingly difficult to manage in areas where agriculture is intense, and the bee’s natural resilience to environmental stress is pushed to a limit (3). Regulators have long warned of a “pollination crisis” that warrants a coordinated response above the level of individual beekeepers (4). In colder climes, including regions of North America, the rate of overwinter colony loss is high enough to routinely trigger public concern in news media, and this rate of loss has renewed interest in monitoring and best practice within the beekeeping community.

The widespread plight of honey bee populations throughout North America likely stems from a mixture of stressors, such as parasitic mites (5), an array of bacterial pathogens (6), pesticides and other environmental toxins (7, 8) as well as habitat loss (3, 9). The beekeeping industry is replete with guidelines on how best to avoid or mitigate some of these stressors. Common among the prescribed remedies is the application of pharmaceutical treatments (e.g., oxytetracycline, tylosin, etc.) against specific pests and pathogens (10). The use of regulated medications can, however, be expensive, impractical, perceived as “unnatural,” and even ineffective if their long-term use inadvertently selects for resistance (11, 12). Antibiotics may also cause dysbiosis and, paradoxically, render colonies more susceptible to secondary infection (13–15). Consequently, the industry seeks new strategies to complement or even replace current practice (16, 17). Ideally, such alternatives will be effective and affordable.

One approach that warrants more testing is the field of microbial therapeutics − that is, the deliberate formulation of microbial supplements that support or enrich the bee’s own native microbiome to beneficial effect (18–21). Unlike antibiotics or other medicated treatments that attack pathogens directly, “probiotics” (22), as applied to beekeeping, complement the host’s health and immune system to help guard against disease and potentially buffer individuals against this and other forms of environmental stress (23). Despite much promise, the effectiveness of probiotics within a commercial beekeeping environment has not been widely tested (24, 25). Previous studies have, however, shown that strains of lactobacilli, among other possibilities, can modulate the immune response, help to assimilate nutrients, and prevent intestinal disorders (26, 27). This lactic acid-producing group of bacteria is therefore a potential source of strains suited for bee-friendly microtherapeutic treatments (28, 29).

A study by our research group tested a combination of three strains – namely, *Lactiplantibacillus plantarum* Lp39, *Apilactobacillus kunkeei* BR-1, and *Lacticaseibacillus rhamnosus* GR-1, which are collectively referred to as “LX3” (30) – for their ability to inhibit growth of *Paenibacillus larvae*, the bacteria that cause American foulbrood disease (31). We showed that supplementation of colonies with LX3 lowered pathogen load in the guts of worker bees and conferred other benefits, including stimulated worker immunity and queen egg laying (13). These studies were, however, limited to a single apiary. New studies have since tested the effectiveness of this (32) or other probiotic formulations (33) at a larger scale. Further, because the application of putatively beneficial bacteria to honey bee colonies is so early in its development, we do not yet know the most effective method of delivery. Intuitively, this would be in edible form, to target the gut directly, but spray-based delivery to large numbers of workers on hive brood frames might also be practical and potentially better suited to ectoparasites like *Varroa* mites that are clearly not situated in the gut. Importantly, using a spray method might more easily keep bacterial cells alive; cells can potentially remain viable for months in phosphate-buffered saline (34). Spray delivery, therefore, offers an alternative way to efficiently distribute living bacteria to a large number of workers within hives (32).

In this study, our objective was to test how delivery of LX3 in patty and spray forms affects pathogen (*P. larvae*) and, for the first time, parasite (*Varroa destructor*) load, and do so across different sites in the Southwestern Ontario (Canada) region.

## MATERIALS AND METHODS

### Culturing

We cultured three strains of lactobacilli: *Lactoplantibacillus plantarum* Lp39 (American Type Culture Collection [ATCC] 14917), *Lactocaseibacillus rhamnosus* GR-1 (ATCC 55826), and *Apilactobacillus kunkeei* BR-1. Briefly, each strain was grown under microaerophilic conditions at 37°C using de Man, Rogosa, and Sharpe (catalog number: 288130, BD Difco) broth or agar supplemented with 10 g/L D-fructose (catalog number: F-3510, Sigma-Aldrich; MRS-F). For both BioPatty- and spray-based LX3 treatments, bacterial cells were collected in a similar manner; following overnight incubation on fresh streak plates, a single colony of each strain was used to inoculate multiple broth cultures, using a separate colony for each culture. The cultures were then uniformly incubated at 37°C for 24 h using sterile 50 mL polypropylene conical tubes (catalog number: 339652, Thermo Scientific; MRS-F filled to 50 mL, lids tightly closed). Cells were collected by centrifugation at 5,000×*g* for 10 min (at 4°C), washed with 0.01 M PBS, and centrifuged again at 5,000×*g* for 10 min (at 4°C). Finally, each strain containing 5 × 10^10^ colony-forming units (CFU) was combined at equal cell densities into a final concentrated volume of 4 mL 0.01 M PBS (phosphate buffer saline solution).

### Patty and spray treatment recipes

We made each 250 g patty using a standard pollen substitute recipe consisting of 28.5 g soy flour, 74.1 g granulated sucrose, 15.4 g debittered brewer’s yeast, and 132.1 g of a simple sucrose-based syrup solution (2:1[w/v]). For the LX3-infused BioPatty (previously described in 30), we added a concentrated suspension of LX3 strains in 0.01 M PBS and mixed until the infusion was visibly homogenous, resulting in a final concentration of 2 × 10^8^ CFU/g for each strain. For vehicle patties, we added an equivalent volume of sterile 0.01 M PBS that did not contain any live bacterial suspension. We followed precedent to make fresh patties (<24 h; reference 32) and store them overnight at 4°C prior to delivery to hives. We positioned each patty between two sheets of wax paper (30 × 45 cm) and, within 24 h of production, placed each patty on top of frames in the brood chamber of Langstroth hives. For the LX3-infused BioSpray (previously described in reference 32), we added the concentrated LX3 suspension to 28 mL of 0.01 M PBS in a sterile spray bottle to obtain a diluted concentration of 1.6 × 10^9^ CFU/mL per strain. As for patties, we prepared the bacteria-infused solution fresh (<24 h) and stored it overnight at 4°C prior to delivery to hives. The nozzle of the bottle discharges 2 mL per spray, so we administered 32 mL of the LX3-containing suspension into the hive via 16 standardized spray actions (2 mL front and 2 mL back of each brood frame, for eight brood frames per hive). The same spray sequence was used for the vehicle spray, but with 32 mL of sterile 0.01 M PBS added to a sterile spray bottle instead.

### Treatment groups, apiary setup, and sampling procedure

**Fig 1:**
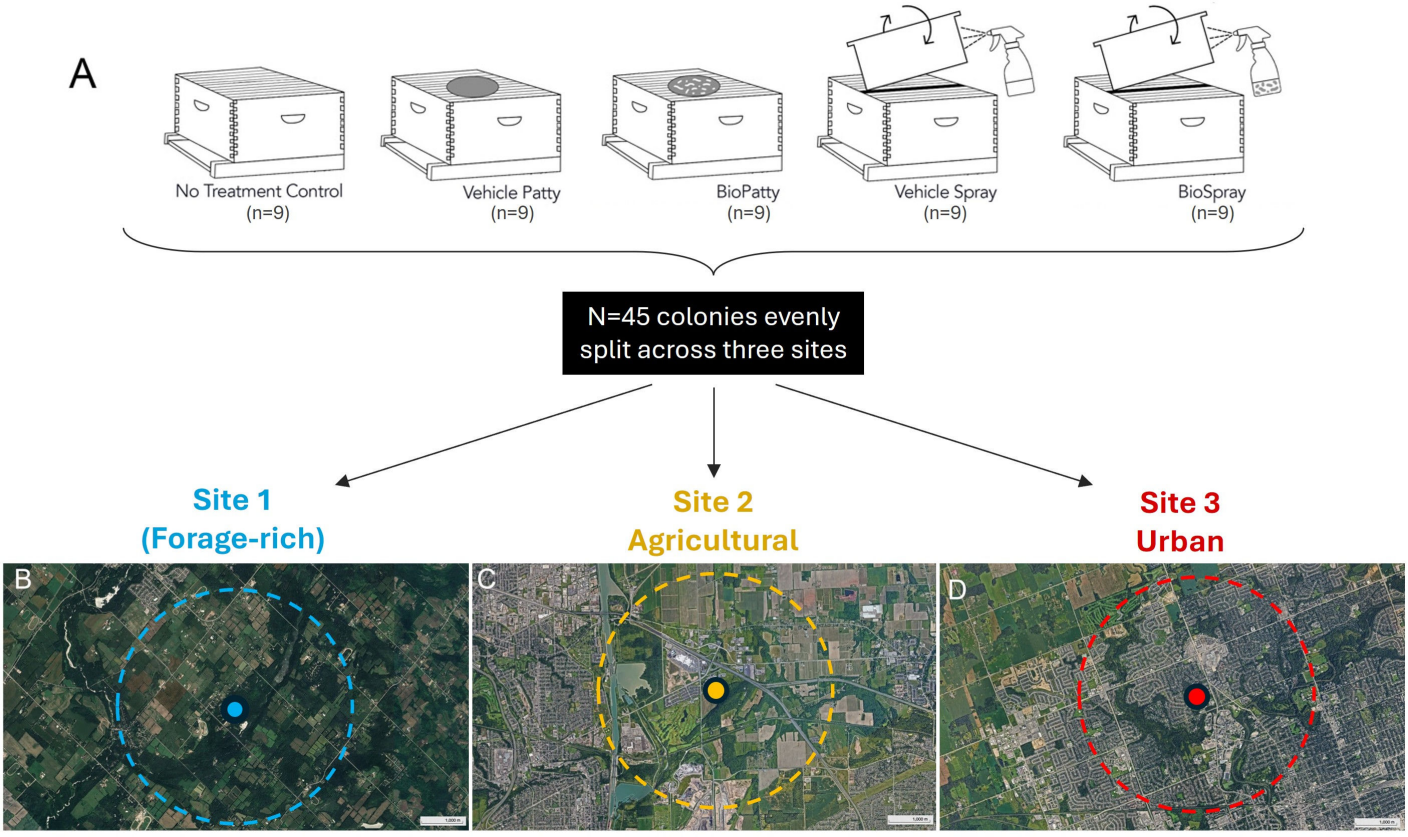
Field study design. (A) There are a total of five treatment groups in this study. They are the no-treatment control, vehicle patty, BioPatty, vehicle spray, BioSpray. Each group was assigned three hives, for a total of *n* = 15 hives at each of three sites (*N* = 45 colonies in total). The sites capture three nominal habitat types in southwestern Ontario: (**B**) forage-rich areas with a high diversity of floral sources, including those with forest patches and feral fields, (**C**) agricultural areas dominated by monoculture crops such as corn and soybean, and (**D**) urban areas near a city with industrial areas and limited forage availability. The dotted lines represent the foraging range of colonies at each site based on the assumption that honey bees typically forage within a 3 km radius from their hives.

The field trials consisted of two treatments (patty and spray) and their respective treatment-specific controls, as well as a full control with no association to treatment or its delivery vehicle (Fig. 1A). We replicated this design across three sites, capturing the response in a naturally forage-rich area (near Milton, ON), a predominantly agricultural site (Niagara-on-the-Lake, ON), and a distinctly urban setting in the City of London, ON (Fig. 1B–D). These land use designations roughly correspond to the “natural land,” “secondary agricultural forage,” and “developed land” of Sobkowich et al.’s (35) Ontario *Varroa* survey.

Within each of the three apiaries, we set up 15 colonies (three colonies × five treatment groups) with new, standard wooden 10-frame Langstroth hive boxes placed on a stand, approximately 12 inches off the ground. We provided each colony with a naturally mated queen of mixed Italian background (*Apis mellifera ligustica*), two frames of capped brood, one frame of honey, one frame of empty wax comb, six empty frames of foundation (for a total of 10 frames per box). In addition, we supplied the equivalent of four full frames worth of bees. All colonies were treated with oxytetracycline 28 days before the start of the experiment. We applied LX3 treatments once a week for eight weeks (July–August 2020). Each colony was equipped with a queen excluder and a medium box with 10 frames above the excluder to allow for additional honey stores as needed. We assigned treatments to colonies at random as: (i) BioPatty group (*n* = 3 hives), (ii) vehicle patty control group (*n* = 3 hives), (iii) BioSpray group (*n* = 3 hives), (iv) vehicle spray control group (*n* = 3 hives), and (v) no treatment control (NTC) group (*n* = 3 hives). A total of 45 colonies were used for the experiment (15 colonies × three locations).

### Estimating *Varroa* load

*Varroa* mite infestation levels were estimated from each colony at weeks 1, 4, and 8 using an alcohol wash technique (36). Briefly, approximately 300 bees were collected from brood frames into a container filled with 70% alcohol. The container was shaken for 2 min to dislodge the mites from the bodies of the worker bees. The mites were then isolated from the wash by filtering through a 1/8 inch mesh wire screen. From mite counts, we estimated the average level of infestation by site and treatment.

### Estimating *P. larvae* load from quantitative PCR of pathogen DNA

To determine the level of *P. larvae* infection in colonies, a total of *n* = 30 adult nurse-age bees (less than 15 days old) were collected from each colony bi-weekly. The bacterial pathogen does not infect the worker caste (it infects larvae; 31) but workers are nonetheless vectors for the bacterial spores and inadvertently infect larvae when tending to them (37). Collected workers were placed into a 50 mL falcon tube and placed on dry ice immediately in the field before transferring to a −80°C freezer to preserve their tissue for molecular analysis. The samples were thawed on ice for 5 min. Then, using forceps, we removed the entire digestive tract by gently pulling on the rectum just above the stinger at the end of the abdomen. Samples that gave the appearance of a pollen-based diet of nurse bees (digestive tracts that were yellow-orange in color) were used for the analysis, while those reflecting a nectar-based diet of forager bees (with an uncolored semi-transparent appearance) were discarded.

DNA was extracted from the samples using a cetyltrimethylammonium bromide (CTAB) method as described previously (37). We used a spectrophotometer to estimate the quality of extracted DNA based on 260/280 and 260/230 ratios of absorbance values. Finally, we used qPCR to quantify pathogen load from bacterial DNA. First, we diluted extracted DNA 10-fold to use as a starting template. Next, we used a SYBR Green-based qPCR kit (Applied Biosystems) with one primer specific to a region of the *16S rRNA* gene of *P. larvae* and another for universal bacterial (341F to 805R) quantification (38, 39), as well as primers specific to the *ribosomal protein S5* gene (40) and the *ß-actin* gene (41) as endogenous controls (Table 1). All qPCR reactions were performed in triplicate in DNase- and RNase-free 384-well plates using routine cycles and a QuantStudio 5 Real-Time PCR System (Applied Biosystems). Amplification data were monitored for “efficiency” and analyzed using QuantStudio Design and Analysis software and used the 2^−ΔΔ^ Ct method to estimate fold-change relative to an endogenous control gene (*ß-actin*).

**TABLE 1:** List of oligonucleotide primers and their target loci. *Beta actin* (host endogenous control gene), *ribosomal protein S5* (host endogenous control gene), universal bacteria (targeting the conserved region 341–805 bp of the *16S rRNA* gene of any bacteria), and *Paenibacillus larvae 16S* (targeting positions 30–407 bp of the *16S rRNA* gene that is specific to *P. larvae*)

Primer target	Reference	Sequence (5′−3′)
*Beta actin*	(41)	F: ATGCCAACACTGTCCTTTCTGG
		R: GACCCACCAATCCATACGGA
*Ribosomal protein S5*	(40)	F: AATTATTTGGTCGCTGGAATTG
		R: TAACGTCCAGCAGAATGTGGTA
*Universal bacteria 16S*	(38)	F: CCTACGGGNGGCWGCAG
		R: GACTACHVGGGTATCTAATCC
*Paenibacillus larvae* 16S	(39)	F: CGGGAGACGCCAGGTTAG
		R: TTCTTCCTTGGCAACAGAGC

### Statistical analysis

Statistical analysis of the data was performed using r (version 4.3.1) and GraphPad (version 10.2.0). For all comparisons, data were analyzed using mixed-effects modeling, whereby treatment (categorical), time (continuous), and their interaction were predictors with location (categorical) as a random variable. A generalized linear mixed model (GLMM) was used to assess global comparisons and emtrends (from the r package emmeans version 1.10.0). We used this analysis to estimate and compare the marginal means of the linear trends. For all comparisons in GraphPad, data were separated by site and analyzed using mixed-effects modelling and Tukey post-hoc test.

## RESULTS

**Fig 2:**
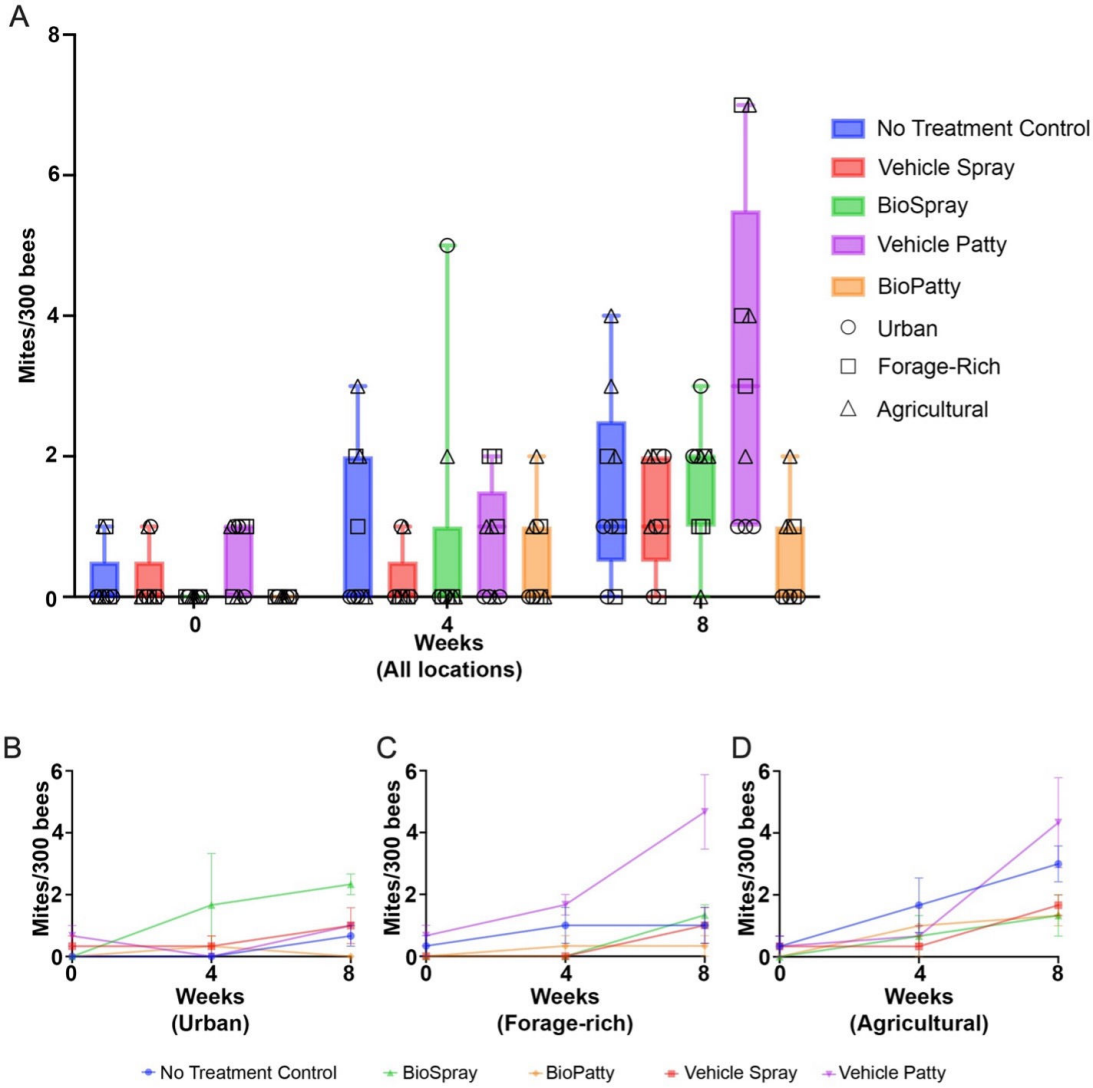
*Varroa* mite count per 300 bees at each timepoint (weeks 0, 4, and 8) for each treatment group. (A) Average mite count per treatment group (*n* = 5 groups) across all three sites at each timepoint (weeks 0, 4, and 8). The data represent the median, inter-quartile range, and minimum/maximum of mite count. Site-specific mite counts for (**B**) urban, (**C**) forage-rich, and (**D**) agricultural habitats.

Baseline levels of parasite and pathogen load were determined for all colonies at each site at the outset of the experiment. Mite populations were estimated at below 1% (0–3 mites per 300 worker bees) on day zero for all colonies in the experiment. A slight increase in mite levels in the NTC group occurred throughout the experiment. Specifically, at day zero, the NTC value was between 0% and 0.33% mite infestation, rising to between 0% and 1.33% after 8 weeks (Fig. 2A). Time had a significant effect on mite levels (*F*_1, 123_ = 45.88, *P* < 0.001), with nearly all colonies showing an increase at the urban, forage-rich, and agricultural settings (Fig. 2B through D).

When looking at the treatment effect, and considering the variation associated with site, time (*F*_1, 123_ = 45.88, *P* < 0.0001) and the interaction between time and treatment (*F*_4, 123_ = 3.01, *P* = 0.021) are significantly different by analysis of variance (ANOVA) (Table S1). This suggests that the effect of treatment on mite infestation changes over time. Further, when we compare slopes of the interaction between time and treatment, there is a significant difference between the BioPatty and vehicle patty groups (T-ratio = −3.25; *P* = 0.013; Table S5), meaning that of all possible pairwise comparisons, these two are the most different.

The forage-rich location was strongly affected by treatment (*F*_4,10_ = 7.859, *P* = 0.004), time (*F*_2,20_ = 17.24, *P* < 0.0001), and their interaction (*F*_8,20_ = 3.856, *P* = 0.007). Here, the vehicle patty had elevated mite loads relative to all other treatments (Fig. 2C), including at 8 weeks in the NTC (*P* < 0.0001), vehicle spray (*P* < 0.0001), BioSpray (*P* = 0.0001), and BioPatty (*P* < 0.0001; Table S3). The overall mite count was also highest at the agricultural site (Fig. 2D), where, again, the vehicle patty had the highest mite loads relative to several other treatments (Table S4). By contrast, the BioPatty treatment resulted in the lowest final mite counts in all three settings (Fig. 2B through D). Indeed, the single greatest difference in mite count was BioPatty vs. vehicle patty at the forage-rich site; the highest mite infestation by week 8 being 0.33% for the BioPatty group and 2.33% for vehicle patty (Fig. 2C). The BioSpray treatment increased the mite count in the urban hives (Fig. 2B).

**Fig 3:**
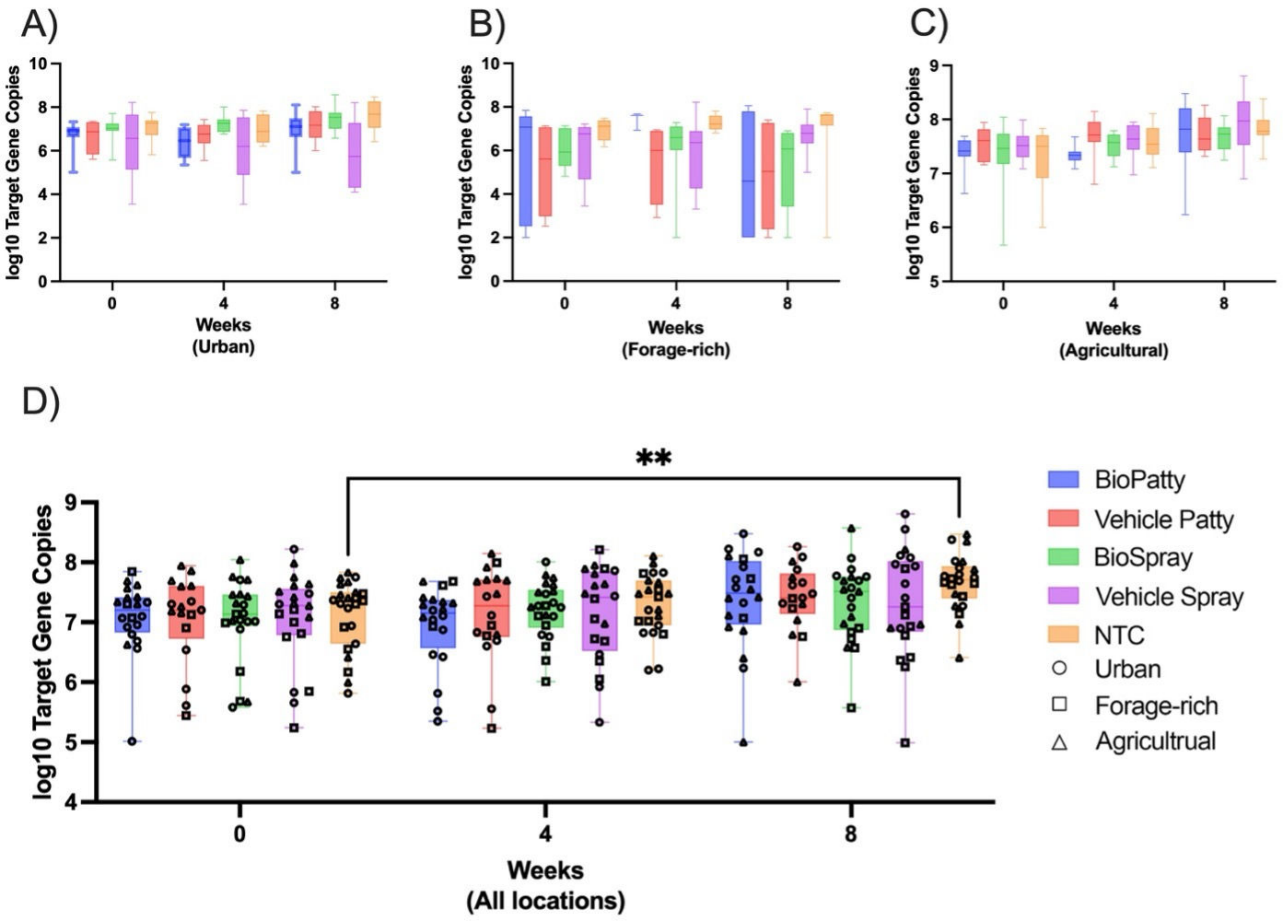
Total bacterial loads over an 8-week period. Total bacterial loads estimated for the (**A**) urban (London), (**B**) forage-rich (Milton), and (**C)** agricultural (Niagara) habitats across three timepoints (weeks 0, 4 and 8). (**D**) Combined total bacterial loads for all three sites. A mixed-effects model and Tukey post-hoc test indicate a significant increase in bacterial load in the no-treatment control group (*P* < 0.005; Tables S1 and S5).

**Fig 4:**
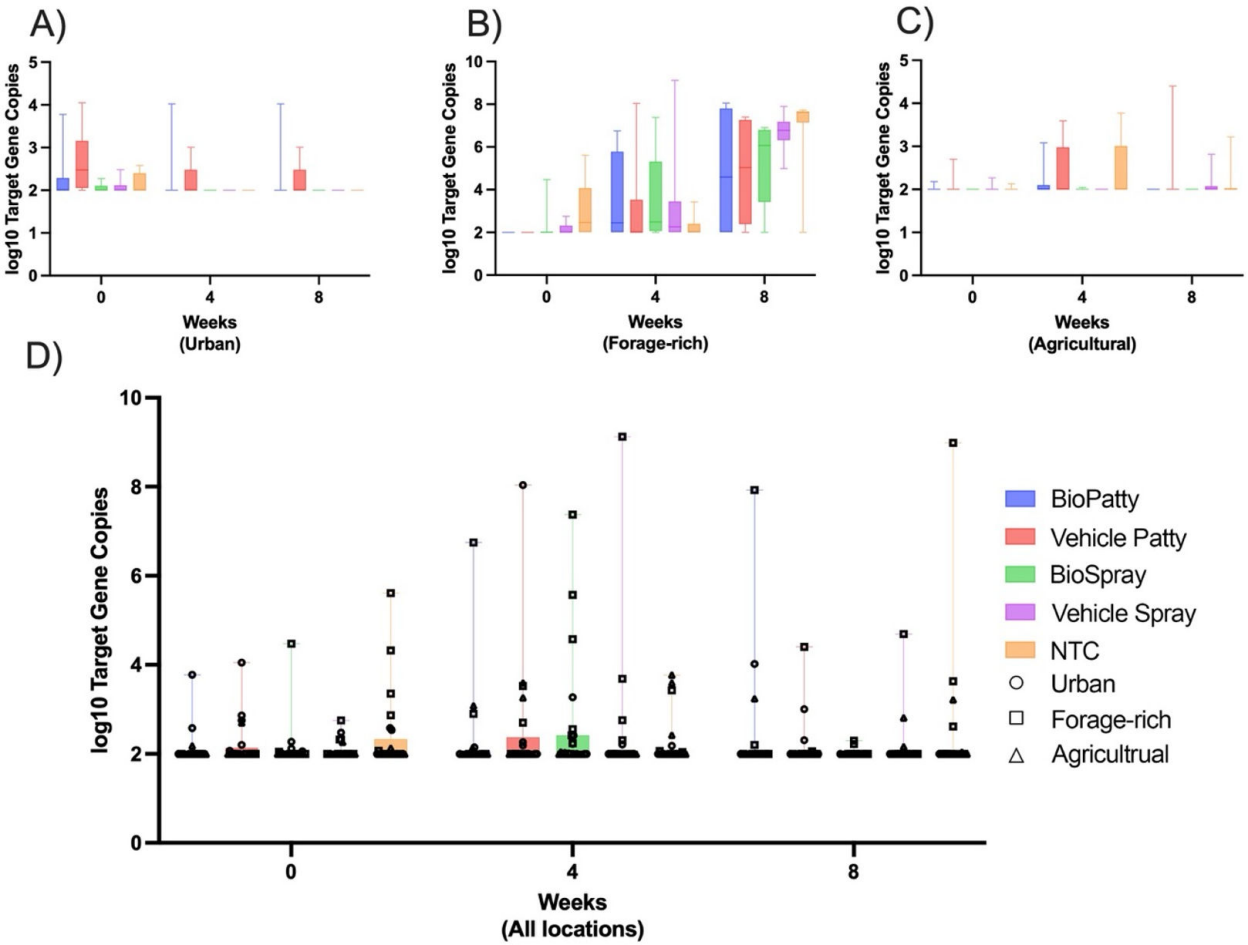
*Paenibacillus larvae* loads over a span of 8 weeks. *P. larvae* load estimated for (**A**) the urban (London), (**B**) forage-rich (Milton), and (**C**) agricultural (Niagara) habitats at weeks 0, 4, and 8. Panel D shows the total pathogenic load summed across all three sites.

For pathogen load, the normalized relative expression of the 16S gene transcript was used to estimate the total bacterial load and specifically the *P. larvae* load. The total bacterial load on a worker bee was consistently ~7 log_10_ gene copies (range, 2.0–8.2 log_10_) throughout all the hives at each setting (*n* = 45) across all timepoints (*n* = 3), while the *P. larvae* load per worker bee was ~2 log_10_ gene copies (range 2.0–4.5). These molecular estimates of bacterial numbers are low and suggest that the asymptomatic colonies in our apiaries had negligible levels of foulbrood infection, and the loads for total bacteria were quite stable over time for all three locations (Fig. 3; Tables S6 to S8), with the forage-rich site being the most variable. Across all treatment groups and sites, *P. larvae* was low or undetectable, with some variability in the forage-rich site (Fig. 4). The American foulbrood-causing bacterium showed no significant difference over time (Tables S9 to S11) or as a function of treatment.

## DISCUSSION

This study used a multi-site field trial to test how patty vs. spray-based delivery of a three-strain consortium of immunostimulatory lactobacilli could influence honey bee resistance to environmental stress, as measured through susceptibility to a specific parasite and pathogen known to afflict managed honey bees in our study region. We showed that the delivery of the LX3 formulation developed by Daisley et al. (30) in the form of a BioPatty was effective at keeping *Varroa* mite loads low, despite a tendency for *Varroa* to increase in untreated control colonies over the course of the experiment. This suppressive effect was observed across three separate sites that reflected local versions of urban, agricultural, and relatively undisturbed naturally forage-rich areas, with the effect being particularly strong at the latter site, near Milton, Ontario. *Varroa* is an ectoparasite that is increasingly common in these regions (35), but our spray-based application of LX3 appeared ineffective in comparison to the patty, which underscores the importance of delivery when applying microbial therapeutics. Both delivery methods do facilitate viable uptake of LX3 in adult bees, although the strains do not appear to colonize long-term (32). Finally, no influence of LX3 on *P. larvae* loads was observed in the asymptomatic colonies studied, which is likely attributable to the already low levels of pathogenic spores and challenges associated with detection limits. Our results therefore show the potential for widespread utility of the lactobacilli-infused BioPatty across a range of beekeeping landscapes.

The level of *Varroa* infestation in our study populations was stable, and for most colonies, it was just below a critical threshold (three or more mites per 100 bees) that usually prompts miticide treatment. The presence of mites in a majority of colonies indicates that there remains a persistent region-wide threat from these devastating, virus-carrying mites in our study region, as first reviewed by Guzmán-Novoa et al. (42). *Varroa* mites are naturally adapted to the Eastern honey bee *Apis cerana* and cause considerably more harm to its newfound host, *Apis mellifera* (43, 44). In our study, mite loads did vary as a function of time and treatment, with the maximum recorded loads nearly quadrupling in no-treatment control colonies (from 1 to 4 mites per 300 sampled bees) over a span of 8 weeks and increasing roughly sevenfold in our vehicle patty group (from 1 to 7 mites per 300 samples bees). The common use of protein patties as a feed supplement to support colony growth (45) may therefore inadvertently increase mite loads, as reported for at least one other study (46).

The undesirable effect of the patty on mite load may be a by-product of increased opportunities for mites to reproduce and develop if, for example, feeding patties to hives results in more drone cells, which are favored by the mites (44, 47). Regardless, this apparent trade-off between colony growth and disease may alert the beekeeping community to limit supplemental feeding with patties in areas where mites are common or, alternatively, formulate patties with the addition of probiotic strains that support the natural resiliency of honey bees against this parasite and other forms of environmental stress. The elevated mite load at the forage-rich site may be related to local variation in bee nutrition, population density, or spatial structuring of the mite, as had been discussed for Ontario (35) and for other *Varroa* surveys (48, 49). The oral administration of LX3 via the BioPatty was helpful against ectoparasites, possibly because of delivery to larvae by worker bees, rendering larvae more resistant, or lactic acid itself may inhibit the mite’s ability to attach or move within the colony (50). Finally, both the BioSpray and vehicle spray sometimes reduced mite levels (in two of three sites), possibly as a by-product of grooming behaviors induced from spraying the honey bees directly with PBS solution. Follow-up studies are now warranted to discern whether the mode of action is via innate or behavioral immunity, or through physiological effects on motor skills.

Our current test of the BioPatty against the bacterial pathogen causing American foulbrood was not strong simply because the presence of *P. larvae* was too light in our colonies to further test this effect. We did, however, notice the *P. larvae* load steadily increased at the forage-rich site, suggesting a latent threat from this pathogen that did not fully manifest during the experiment. Previous work has shown it can be difficult to induce or detect effects of bacterial supplementation in field colonies (51, 52). In our case, we simply did not have enough disease to suppress, so any colony-level effect our treatment might have had under higher pathogen loads is unknown. The low baseline level of *P. larvae* is probably due to the condition of our colonies, which began as nucleus colonies in entirely new equipment. Previous studies have, however, shown that the BioPatty can temporarily alter the gut microbiome (32), help to control *P. larvae* (30), and help to restore the gut microbiome following antibiotic perturbation (13). BioSpray, by contrast, may nonetheless be effective at controlling microbial brood pathogens like *Melissococcus plutonius* and *Ascosphaera apis* (32). This leaves beekeepers with a dilemma since the LX3 formulation is not commercially available in patty or spray form, and alternative commercial “probiotic” products are not typically tested to prove their effectiveness (21, 24, 25). Nonetheless, microbial therapeutics can leverage the bee’s natural abilities to stave off some stress via the gut-microbe barrier (53).

The application of probiotics as a strategic treatment to colonies in stress has the potential to become an integral component of a comprehensive disease management strategy. Based on current research, we advocate for both patty and spray-based delivery mechanisms that involve LX3 or other therapies shown to be effective (e.g., 54). In each case, however, clear protocols need to be established that link to a beekeeper’s specific needs so that potentially useful therapies are not administered incorrectly or in vain. The implications of microbial therapeutic research include the possibility of enhancing the resilience of managed honey bee colonies against diseases and thus helping to future-proof apiculture and pollination services.

## SUPPLEMENTAL MATERIAL

Supplemental material spectrum.02498-24-s0001.docx Tables S1 to S18.

ASM does not own the copyrights to Supplemental Material that may be linked to, or accessed through, an article. The authors have granted ASM a non-exclusive, world-wide license to publish the Supplemental Material files. Please contact the corresponding author directly for reuse.
