# Supplementary material for: Probiotic effects on ectoparasitic mite infestations in honey bees (Apis mellifera) are modulated by environmental conditions and route of administration
Source: Microbiol Spectr. 2025 May 22;13(7):e02498-24. doi: 10.1128/spectrum.02498-24 (PMC12210957; doi:10.1128/spectrum.02498-24)
Supplement: Supplemental material — Tables S1 to S18. [file spectrum.02498-24-s0001.docx]

**Supplementary Tables**

| **Table S1.** Two-way analysis of variance (two-way ANOVA) for mite infestation across three sites, three timepoints (weeks 0, 3, 8), and treatments (n=5 groups). | | | | | |
| --- | --- | --- | --- | --- | --- |
| **Source of Variation** | **SS** | **DF** | **MS** | **F _(DFn, DFd)_** | ***P* value** |
| Treatment | 0.382 | 4 | 0.960 | F_4, 123_ = 0.906 | *P* = 0.985 |
| Time (Weeks) | 48.400 | 1 | 48.400 | F_1, 123_ = 45.88 | *P* < 0.0001 |
| Interaction | 12.711 | 4 | 3.178 | F_4, 123_ = 3.013 | *P* = 0.0201 |

**Table S2.** Two-way analysis of variance (tweo-way ANOVA) and Tukey’s multiple comparisons test of *Varroa destructor* mite infestation levels for all five treatment groups (n=3 hives per group) at each week (n=3 timepoints) for the urban location. Tukey HSD test results are bolded for any significant difference between treatment groups within a single timepoint.

|  | **Tukey's multiple comparisons test** | **Mean Diff.** | **95.00% CI of diff.** | **Adjusted *P* value** |
| --- | --- | --- | --- | --- |
| Week 0 | No Treatment Control vs. Vehicle Spray | -0.333 | -2.392 to 1.725 | 0.990 |
|  | No Treatment Control vs. BioSpray | 0.000 | -2.059 to 2.059 | 0.999 |
|  | No Treatment Control vs. Vehicle Patty | -0.667 | -2.725 to 1.392 | 0.879 |
|  | No Treatment Control vs. BioPatty | 0.000 | -2.059 to 2.059 | 0.999 |
|  | Vehicle Spray vs. BioSpray | 0.333 | -1.725 to 2.392 | 0.990 |
|  | Vehicle Spray vs. Vehicle Patty | -0.333 | -2.392 to 1.725 | 0.990 |
|  | Vehicle Spray vs. BioPatty | 0.333 | -1.725 to 2.392 | 0.990 |
|  | BioSpray vs. Vehicle Patty | -0.667 | -2.725 to 1.392 | 0.879 |
|  | BioSpray vs. BioPatty | 0.000 | -2.059 to 2.059 | 0.999 |
|  | Vehicle Patty vs. BioPatty | 0.667 | -1.392 to 2.725 | 0.879 |
| Week 4 | No Treatment Control vs. Vehicle Spray | -0.3333 | -2.392 to 1.725 | 0.990 |
|  | No Treatment Control vs. BioSpray | -1.667 | -3.725 to 0.3920 | 0.158 |
|  | No Treatment Control vs. Vehicle Patty | 0.000 | -2.059 to 2.059 | 0.999 |
|  | No Treatment Control vs. BioPatty | -0.3333 | -2.392 to 1.725 | 0.990 |
|  | Vehicle Spray vs. BioSpray | -1.333 | -3.392 to 0.7253 | 0.350 |
|  | Vehicle Spray vs. Vehicle Patty | 0.3333 | -1.725 to 2.392 | 0.990 |
|  | Vehicle Spray vs. BioPatty | 3.331e-016 | -2.059 to 2.059 | 0.999 |
|  | BioSpray vs. Vehicle Patty | 1.667 | -0.3920 to 3.725 | 0.158 |
|  | BioSpray vs. BioPatty | 1.333 | -0.7253 to 3.392 | 0.350 |
|  | Vehicle Patty vs. BioPatty | -0.3333 | -2.392 to 1.725 | 0.990 |
| Week 8 | No Treatment Control vs. Vehicle Spray | -0.3333 | -2.392 to 1.725 | 0.990 |
|  | No Treatment Control vs. BioSpray | -1.667 | -3.725 to 0.3920 | 0.159 |
|  | No Treatment Control vs. Vehicle Patty | -0.3333 | -2.392 to 1.725 | 0.990 |
|  | No Treatment Control vs. BioPatty | 0.6667 | -1.392 to 2.725 | 0.880 |
|  | Vehicle Spray vs. BioSpray | -1.333 | -3.392 to 0.7253 | 0.350 |
|  | Vehicle Spray vs. Vehicle Patty | 0.000 | -2.059 to 2.059 | 0.999 |
|  | Vehicle Spray vs. BioPatty | 1.000 | -1.059 to 3.059 | 0.627 |
|  | BioSpray vs. Vehicle Patty | 1.333 | -0.7253 to 3.392 | 0.350 |
|  | BioSpray vs. BioPatty | 2.333 | 0.2747 to 4.392 | **0.020** |
|  | Vehicle Patty vs. BioPatty | 1.000 | -1.059 to 3.059 | 0.627 |

**Table S3.**  Two-way analysis of variance (ANOVA) and Tukey’s multiple comparisons test of *Varroa destructor* mite infestation levels for all five treatment groups (n=3 hives per group) at each week (n=3 timepoints) for the forage-rich location. Tukey HSD test results are bolded for any significant difference between treatment groups within a single timepoint.

|  | **Tukey's multiple comparisons test** | **Mean Diff.** | **95.00% CI of diff.** | **Adjusted *P* value** |
| --- | --- | --- | --- | --- |
| Week 0 | No Treatment Control vs. Vehicle Spray | 0.333 | -1.535 to 2.202 | 0.985 |
|  | No Treatment Control vs. BioSpray | 0.333 | -1.535 to 2.202 | 0.985 |
|  | No Treatment Control vs. Vehicle Patty | -0.333 | -2.202 to 1.535 | 0.985 |
|  | No Treatment Control vs. BioPatty | 0.333 | -1.535 to 2.202 | 0.985 |
|  | Vehicle Spray vs. BioSpray | 0.000 | -1.868 to 1.868 | 0.999 |
|  | Vehicle Spray vs. Vehicle Patty | -0.667 | -2.535 to 1.202 | 0.837 |
|  | Vehicle Spray vs. BioPatty | 0.000 | -1.868 to 1.868 | 0.999 |
|  | BioSpray vs. Vehicle Patty | -0.667 | -2.535 to 1.202 | 0.837 |
|  | BioSpray vs. BioPatty | 0.000 | -1.868 to 1.868 | 0.999 |
|  | Vehicle Patty vs. BioPatty | 0.667 | -1.202 to 2.535 | 0.837 |
| Week 4 | No Treatment Control vs. Vehicle Spray | 1.000 | -0.8682 to 2.868 | 0.538 |
|  | No Treatment Control vs. BioSpray | 1.000 | -0.8682 to 2.868 | 0.538 |
|  | No Treatment Control vs. Vehicle Patty | -0.667 | -2.535 to 1.202 | 0.837 |
|  | No Treatment Control vs. BioPatty | 0.667 | -1.202 to 2.535 | 0.837 |
|  | Vehicle Spray vs. BioSpray | 0.000 | -1.868 to 1.868 | 0.999 |
|  | Vehicle Spray vs. Vehicle Patty | -1.667 | -3.535 to 0.2015 | 0.098 |
|  | Vehicle Spray vs. BioPatty | -0.333 | -2.202 to 1.535 | 0.985 |
|  | BioSpray vs. Vehicle Patty | -1.667 | -3.535 to 0.2015 | 0.098 |
|  | BioSpray vs. BioPatty | -0.333 | -2.202 to 1.535 | 0.985 |
|  | Vehicle Patty vs. BioPatty | 1.333 | -0.5348 to 3.202 | 0.259 |
| Week 8 | No Treatment Control vs. Vehicle Spray | 0.000 | -1.868 to 1.868 | 0.999 |
|  | No Treatment Control vs. BioSpray | -0.333 | -2.202 to 1.535 | 0.985 |
|  | No Treatment Control vs. Vehicle Patty | -3.667 | -5.535 to -1.798 | **<0.0001** |
|  | No Treatment Control vs. BioPatty | 0.667 | -1.202 to 2.535 | 0.837 |
|  | Vehicle Spray vs. BioSpray | -0.333 | -2.202 to 1.535 | 0.985 |
|  | Vehicle Spray vs. Vehicle Patty | -3.667 | -5.535 to -1.798 | **<0.0001** |
|  | Vehicle Spray vs. BioPatty | 0.667 | -1.202 to 2.535 | 0.837 |
|  | BioSpray vs. Vehicle Patty | -3.333 | -5.202 to -1.465 | **0.0001** |
|  | BioSpray vs. BioPatty | 1.000 | -0.8682 to 2.868 | 0.538 |
|  | Vehicle Patty vs. BioPatty | 4.333 | 2.465 to 6.202 | **<0.0001** |

**Table S4.** Two-way analysis of variance (ANOVA) and Tukey’s multiple comparisons test of *Varroa destructor* mite infestation levels for all five treatment groups (n=3 hives per group) at each week (n=3 timepoints) for the agricultural location. Tukey HSD test results are bolded for any significant difference between treatment groups within a single timepoint.

|  | **Tukey's multiple comparisons test** | **Mean Diff.** | **95.00% CI of diff.** | **Adjusted *P* value** |
| --- | --- | --- | --- | --- |
| Week 0 | No Treatment Control vs. Vehicle Spray | -2.220e-016 | -2.420 to 2.420 | 0.999 |
|  | No Treatment Control vs. BioSpray | 0.333 | -2.087 to 2.754 | 0.994 |
|  | No Treatment Control vs. Vehicle Patty | -2.220e-016 | -2.420 to 2.420 | 0.999 |
|  | No Treatment Control vs. BioPatty | 0.333 | -2.087 to 2.754 | 0.994 |
|  | Vehicle Spray vs. BioSpray | 0.333 | -2.087 to 2.754 | 0.994 |
|  | Vehicle Spray vs. Vehicle Patty | 0.000 | -2.420 to 2.420 | 0.999 |
|  | Vehicle Spray vs. BioPatty | 0.333 | -2.087 to 2.754 | 0.994 |
|  | BioSpray vs. Vehicle Patty | -0.333 | -2.754 to 2.087 | 0.994 |
|  | BioSpray vs. BioPatty | 0.000 | -2.420 to 2.420 | 0.999 |
|  | Vehicle Patty vs. BioPatty | 0.333 | -2.087 to 2.754 | 0.994 |
| Week 4 | No Treatment Control vs. Vehicle Spray | 1.333 | -1.087 to 3.754 | 0.510 |
|  | No Treatment Control vs. BioSpray | 1.000 | -1.420 to 3.420 | 0.752 |
|  | No Treatment Control vs. Vehicle Patty | 1.000 | -1.420 to 3.420 | 0.752 |
|  | No Treatment Control vs. BioPatty | 0.667 | -1.754 to 3.087 | 0.929 |
|  | Vehicle Spray vs. BioSpray | -0.333 | -2.754 to 2.087 | 0.994 |
|  | Vehicle Spray vs. Vehicle Patty | -0.333 | -2.754 to 2.087 | 0.994 |
|  | Vehicle Spray vs. BioPatty | -0.667 | -3.087 to 1.754 | 0.929 |
|  | BioSpray vs. Vehicle Patty | 0.000 | -2.420 to 2.420 | 0.999 |
|  | BioSpray vs. BioPatty | -0.333 | -2.754 to 2.087 | 0.994 |
|  | Vehicle Patty vs. BioPatty | -0.333 | -2.754 to 2.087 | 0.994 |
| Week 8 | No Treatment Control vs. Vehicle Spray | 1.333 | -1.087 to 3.754 | 0.510 |
|  | No Treatment Control vs. BioSpray | 1.667 | -0.7537 to 4.087 | 0.292 |
|  | No Treatment Control vs. Vehicle Patty | -1.333 | -3.754 to 1.087 | 0.510 |
|  | No Treatment Control vs. BioPatty | 1.667 | -0.7537 to 4.087 | 0.292 |
|  | Vehicle Spray vs. BioSpray | 0.333 | -2.087 to 2.754 | 0.994 |
|  | Vehicle Spray vs. Vehicle Patty | -2.667 | -5.087 to -0.2463 | **0.025** |
|  | Vehicle Spray vs. BioPatty | 0.333 | -2.087 to 2.754 | 0.994 |
|  | BioSpray vs. Vehicle Patty | -3.000 | -5.420 to -0.5796 | **0.009** |
|  | BioSpray vs. BioPatty | 0.000 | -2.420 to 2.420 | 0.999 |
|  | Vehicle Patty vs. BioPatty | 3.000 | 0.5796 to 5.420 | **0.009** |

**Table S5.** Pairwise comparisons of estimated marginal mean of the slope of treatment groups for *Varroa destructor* mite infestation levels. Confidence level used: 0.95; *P*-value adjustment: Tukey method for comparing a family of five estimates.

| **Comparison** | **Estimate** | **SE** | **df** | **T-ratio** | **Adjusted *P* value** |
| --- | --- | --- | --- | --- | --- |
| NTC vs. BioPatty | 0.097 | 0.086 | 123 | 1.136 | 0.787 |
| NTC vs. BioSpray | -0.042 | 0.086 | 123 | -0.487 | 0.988 |
| NTC vs. Vehicle Patty | -0.181 | 0.086 | 123 | -2.11 | 0.223 |
| NTC vs. Vehicle Spray | 0.042 | 0.086 | 123 | 0.487 | 0.988 |
| BioPatty vs. BioSpray | -0.139 | 0.086 | 123 | -1.623 | 0.486 |
| BioPatty vs. Vehicle Patty | -0.278 | 0.086 | 123 | -3.246 | **0.013** |
| BioPatty vs. Vehicle Spray | -0.056 | 0.086 | 123 | -0.649 | 0.967 |
| BioSpray vs. Vehicle Patty | -0.139 | 0.086 | 123 | -1.623 | 0.486 |
| BioSpray vs. Vehicle Spray | 0.083 | 0.086 | 123 | 0.974 | 0.867 |
| Vehicle Patty vs. Vehicle Spray | 0.016 | 0.086 | 123 | 2.597 | 0.077 |

**Table S6.** Two-way analysis of variance (ANOVA) and Tukey’s multiple comparisons test of total bacterial loads for all five treatment groups (n=3 hives per group) at each week (n=3 timepoints) for the urban location. Tukey HSD test results are bolded for any significant difference between treatment groups within a single timepoint.

|  | **Tukey's multiple comparisons test** | **Mean Diff.** | **95.00% CI of diff.** | **Adjusted *P* value** |
| --- | --- | --- | --- | --- |
| Week 0 | BioPatty vs. Vehicle Patty | 0.067 | -1.252 to 1.386 | 0.999 |
|  | BioPatty vs. BioSpray | -0.244 | -1.460 to 0.9723 | 0.981 |
|  | BioPatty vs. Vehicle Spray | 0.377 | -0.9424 to 1.696 | 0.932 |
|  | BioPatty vs. NTC | -0.343 | -1.559 to 0.8730 | 0.935 |
|  | Vehicle Patty vs. BioSpray | -0.310 | -1.662 to 1.041 | 0.968 |
|  | Vehicle Patty vs. Vehicle Spray | 0.310 | -1.135 to 1.755 | 0.975 |
|  | Vehicle Patty vs. NTC | -0.410 | -1.761 to 0.9420 | 0.917 |
|  | BioSpray vs. Vehicle Spray | 0.620 | -0.7313 to 1.972 | 0.707 |
|  | BioSpray vs. NTC | -0.099 | -1.351 to 1.152 | 1.000 |
|  | Vehicle Spray vs. NTC | -0.720 | -2.071 to 0.6320 | 0.578 |
| Week 4 | BioPatty vs. Vehicle Patty | -0.285 | -1.604 to 1.034 | 0.975 |
|  | BioPatty vs. BioSpray | -0.827 | -2.043 to 0.3894 | 0.330 |
|  | BioPatty vs. Vehicle Spray | 0.328 | -0.9912 to 1.647 | 0.958 |
|  | BioPatty vs. NTC | -0.565 | -1.781 to 0.6513 | 0.697 |
|  | Vehicle Patty vs. BioSpray | -0.541 | -1.893 to 0.8103 | 0.799 |
|  | Vehicle Patty vs. Vehicle Spray | 0.613 | -0.8317 to 2.058 | 0.763 |
|  | Vehicle Patty vs. NTC | -0.279 | -1.631 to 1.072 | 0.979 |
|  | BioSpray vs. Vehicle Spray | 1.154 | -0.1972 to 2.506 | 0.131 |
|  | BioSpray vs. NTC | 0.262 | -0.9894 to 1.513 | 0.977 |
|  | Vehicle Spray vs. NTC | -0.892 | -2.244 to 0.4591 | 0.359 |
| Week 8 | BioPatty vs. Vehicle Patty | -0.177 | -1.496 to 1.142 | 0.996 |
|  | BioPatty vs. BioSpray | -0.492 | -1.708 to 0.7237 | 0.793 |
|  | BioPatty vs. Vehicle Spray | 1.122 | -0.1972 to 2.441 | 0.134 |
|  | BioPatty vs. NTC | -0.630 | -1.846 to 0.5860 | 0.603 |
|  | Vehicle Patty vs. BioSpray | -0.315 | -1.666 to 1.037 | 0.967 |
|  | Vehicle Patty vs. Vehicle Spray | 1.299 | -0.1458 to 2.744 | 0.099 |
|  | Vehicle Patty vs. NTC | -0.453 | -1.804 to 0.8989 | 0.884 |
|  | BioSpray vs. Vehicle Spray | 1.614 | 0.2625 to 2.966 | **0.011** |
|  | BioSpray vs. NTC | -0.138 | -1.389 to 1.114 | 0.998 |
|  | Vehicle Spray vs. NTC | -1.752 | -3.103 to -0.4002 | **0.005** |

**Table S7.** Two-way analysis of variance (ANOVA) and Tukey’s multiple comparisons test of total bacterial loads for all five treatment groups (n=3 hives per group) at each week (n=3 timepoints) for the forage-rich location. Tukey HSD test results are bolded for any significant difference between treatment groups within a single timepoint.

|  | **Tukey's multiple comparisons test** | **Mean Diff.** | **95.00% CI of diff.** | **Adjusted *P* value** |
| --- | --- | --- | --- | --- |
| Week 0 | BioPatty vs. Vehicle Patty | 0.230 | -5.646 to 6.105 | 0.999 |
|  | BioPatty vs. BioSpray | -0.594 | -5.808 to 4.620 | 0.987 |
|  | BioPatty vs. Vehicle Spray | -0.500 | -5.641 to 4.641 | 0.994 |
|  | BioPatty vs. NTC | -1.515 | -6.844 to 3.813 | 0.741 |
|  | Vehicle Patty vs. BioSpray | -0.823 | -6.226 to 4.579 | 0.943 |
|  | Vehicle Patty vs. Vehicle Spray | -0.730 | -5.966 to 4.506 | 0.966 |
|  | Vehicle Patty vs. NTC | -1.745 | -7.411 to 3.921 | 0.595 |
|  | BioSpray vs. Vehicle Spray | 0.094 | -1.786 to 1.974 | 1.000 |
|  | BioSpray vs. NTC | -0.922 | -2.396 to 0.5532 | 0.283 |
|  | Vehicle Spray vs. NTC | -1.015 | -2.681 to 0.6503 | 0.338 |
| Week 4 | BioPatty vs. Vehicle Patty | 1.940 | -2.763 to 6.643 | 0.411 |
|  | BioPatty vs. BioSpray | 1.366 | -1.240 to 3.972 | 0.412 |
|  | BioPatty vs. Vehicle Spray | 1.535 | -0.4191 to 3.489 | 0.147 |
|  | BioPatty vs. NTC | 0.140 | -1.173 to 1.453 | 0.984 |
|  | Vehicle Patty vs. BioSpray | -0.574 | -4.867 to 3.720 | 0.985 |
|  | Vehicle Patty vs. Vehicle Spray | -0.405 | -4.678 to 3.868 | 0.994 |
|  | Vehicle Patty vs. NTC | -1.800 | -6.623 to 3.023 | 0.456 |
|  | BioSpray vs. Vehicle Spray | 0.169 | -2.632 to 2.970 | 1.000 |
|  | BioSpray vs. NTC | -1.226 | -3.815 to 1.363 | 0.481 |
|  | Vehicle Spray vs. NTC | -1.395 | -3.279 to 0.4894 | 0.178 |
| Week 8 | BioPatty vs. Vehicle Patty | -0.040 | -5.418 to 5.337 | 0.999 |
|  | BioPatty vs. BioSpray | -0.408 | -5.350 to 4.534 | 0.998 |
|  | BioPatty vs. Vehicle Spray | -1.839 | -6.809 to 3.130 | 0.634 |
|  | BioPatty vs. NTC | -1.920 | -6.954 to 3.114 | 0.702 |
|  | Vehicle Patty vs. BioSpray | -0.368 | -4.501 to 3.765 | 0.998 |
|  | Vehicle Patty vs. Vehicle Spray | -1.799 | -5.860 to 2.263 | 0.504 |
|  | Vehicle Patty vs. NTC | -1.880 | -6.174 to 2.414 | 0.616 |
|  | BioSpray vs. Vehicle Spray | -1.431 | -3.858 to 0.9966 | 0.348 |
|  | BioSpray vs. NTC | -1.512 | -4.821 to 1.797 | 0.609 |
|  | Vehicle Spray vs. NTC | -0.081 | -3.042 to 2.879 | 0.999 |

**Table S8.** Two-way analysis of variance (ANOVA) and Tukey’s multiple comparisons test of total bacterial loads for all five treatment groups (n=3 hives per group) at each week (n=3 timepoints) for the agricultural location. Tukey HSD tests results are bolded for significant difference between treatment groups within a single timepoint.

|  | **Tukey's multiple comparisons test** | **Mean Diff.** | **95.00% CI of diff.** | **Adjusted *P* value** |
| --- | --- | --- | --- | --- |
| Week 0 | BioPatty vs. Vehicle Patty | -0.167 | -0.662 to 0.328 | 0.827 |
|  | BioPatty vs. BioSpray | 0.052 | -0.765 to 0.868 | 1.000 |
|  | BioPatty vs. Vehicle Spray | -0.138 | -0.601 to 0.325 | 0.879 |
|  | BioPatty vs. NTC | 0.078 | -0.697 to 0.852 | 0.997 |
|  | Vehicle Patty vs. BioSpray | 0.219 | -0.591 to 1.028 | 0.902 |
|  | Vehicle Patty vs. Vehicle Spray | 0.029 | -0.408 to 0.466 | 0.999 |
|  | Vehicle Patty vs. NTC | 0.245 | -0.521 to 1.011 | 0.840 |
|  | BioSpray vs. Vehicle Spray | -0.190 | -0.988 to 0.609 | 0.932 |
|  | BioSpray vs. NTC | 0.026 | -0.931 to 0.983 | 0.999 |
|  | Vehicle Spray vs. NTC | 0.216 | -0.538 to 0.970 | 0.880 |
| Week 4 | BioPatty vs. Vehicle Patty | -0.341 | -0.800 to 0.117 | 0.185 |
|  | BioPatty vs. BioSpray | -0.183 | -0.5054to 0.140 | 0.419 |
|  | BioPatty vs. Vehicle Spray | -0.285 | -0.6660to 0.095 | 0.186 |
|  | BioPatty vs. NTC | -0.243 | -0.635 to 0.150 | 0.337 |
|  | Vehicle Patty vs. BioSpray | 0.158 | -0.322 to 0.638 | 0.836 |
|  | Vehicle Patty vs. Vehicle Spray | 0.056 | -0.455 to 0.566 | 0.997 |
|  | Vehicle Patty vs. NTC | 0.098 | -0.419 to 0.616 | 0.975 |
|  | BioSpray vs. Vehicle Spray | -0.102 | -0.514 to 0.309 | 0.935 |
|  | BioSpray vs. NTC | -0.060 | -0.482 to 0.362 | 0.991 |
|  | Vehicle Spray vs. NTC | 0.043 | -0.417 to 0.502 | 0.998 |
| Week 8 | BioPatty vs. Vehicle Patty | -0.007 | -0.912 to 0.897 | 0.999 |
|  | BioPatty vs. BioSpray | 0.030 | -0.862 to 0.922 | 0.999 |
|  | BioPatty vs. Vehicle Spray | -0.228 | -1.208 to 0.753 | 0.947 |
|  | BioPatty vs. NTC | -0.116 | -1.016 to 0.784 | 0.992 |
|  | Vehicle Patty vs. BioSpray | 0.037 | -0.436 to 0.510 | 0.999 |
|  | Vehicle Patty vs. Vehicle Spray | -0.220 | -0.928 to 0.488 | 0.860 |
|  | Vehicle Patty vs. NTC | -0.109 | -0.615 to 0.398 | 0.960 |
|  | BioSpray vs. Vehicle Spray | -0.257 | -0.940 to 0.425 | 0.746 |
|  | BioSpray vs. NTC | -0.146 | -0.598 to 0.306 | 0.847 |
|  | Vehicle Spray vs. NTC | 0.112 | -0.587 to 0.811 | 0.985 |

**Table S9.** Two-way analysis of variance (ANOVA) and Tukey’s multiple comparisons test of *P. larvae* loads for all five treatment groups (n=3 hives per group) at each week (n=3 timepoints) for the urban location. Tukey HSD test results are bolded for any significant difference between treatment groups within a single timepoint.

|  | **Tukey's multiple comparisons test** | **Mean Diff.** | **95.00% CI of diff.** | **Adjusted *P* value** |
| --- | --- | --- | --- | --- |
| Week 0 | BioPatty vs. Vehicle Patty | -0.394 | -0.982 to 0.195 | 0.346 |
|  | BioPatty vs. BioSpray | 0.205 | -0.337 to 0.748 | 0.830 |
|  | BioPatty vs. Vehicle Spray | 0.182 | -0.407 to 0.771 | 0.911 |
|  | BioPatty vs. NTC | 0.123 | -0.420 to 0.665 | 0.970 |
|  | Vehicle Patty vs. BioSpray | 0.599 | -0.004 to 1.203 | 0.052 |
|  | Vehicle Patty vs. Vehicle Spray | 0.576 | -0.069 to 1.221 | 0.103 |
|  | Vehicle Patty vs. NTC | 0.517 | -0.087 to 1.120 | 0.130 |
|  | BioSpray vs. Vehicle Spray | -0.023 | -0.626 to 0.580 | 0.999 |
|  | BioSpray vs. NTC | -0.083 | -0.641 to 0.476 | 0.994 |
|  | Vehicle Spray vs. NTC | -0.060 | -0.663 to 0.544 | 0.999 |
| Week 4 | BioPatty vs. Vehicle Patty | 0.006 | -0.583 to 0.594 | 0.999 |
|  | BioPatty vs. BioSpray | 0.225 | -0.318 to 0.767 | 0.779 |
|  | BioPatty vs. Vehicle Spray | 0.225 | -0.364 to 0.813 | 0.826 |
|  | BioPatty vs. NTC | 0.225 | -0.318 to 0.767 | 0.779 |
|  | Vehicle Patty vs. BioSpray | 0.219 | -0.384 to 0.822 | 0.851 |
|  | Vehicle Patty vs. Vehicle Spray | 0.219 | -0.426 to 0.864 | 0.879 |
|  | Vehicle Patty vs. NTC | 0.219 | -0.384 to 0.822 | 0.851 |
|  | BioSpray vs. Vehicle Spray | 0.000 | -0.603 to 0.603 | 0.999 |
|  | BioSpray vs. NTC | 0.000 | -0.559 to 0.559 | 0.999 |
|  | Vehicle Spray vs. NTC | 0.000 | -0.603 to 0.603 | 0.999 |
| Week 8 | BioPatty vs. Vehicle Patty | 0.006 | -0.583 to 0.594 | 0.999 |
|  | BioPatty vs. BioSpray | 0.225 | -0.318 to 0.767 | 0.779 |
|  | BioPatty vs. Vehicle Spray | 0.225 | -0.364 to 0.813 | 0.826 |
|  | BioPatty vs. NTC | 0.225 | -0.318 to 0.767 | 0.779 |
|  | Vehicle Patty vs. BioSpray | 0.219 | -0.384 to 0.822 | 0.851 |
|  | Vehicle Patty vs. Vehicle Spray | 0.219 | -0.423 to 0.864 | 0.879 |
|  | Vehicle Patty vs. NTC | 0.219 | -0.384 to 0.822 | 0.851 |
|  | BioSpray vs. Vehicle Spray | 0.000 | -0.603 to 0.603 | 0.999 |
|  | BioSpray vs. NTC | 0.000 | -0.559 to 0.559 | 0.999 |
|  | Vehicle Spray vs. NTC | 0.000 | -0.603 to 0.603 | 0.999 |

**Table S10.** Two-way analysis of variance (ANOVA) and Tukey’s multiple comparisons test of *P. larvae* loads for all five treatment groups (n=3 hives per group) at each week (n=3 timepoints) for the forage-rich location. Tukey HSD tests results are bolded for significant difference between treatment groups within a single timepoint.

|  | **Tukey's multiple comparisons test** | **Mean Diff.** | **95.00% CI of diff.** | **Adjusted *P* value** |
| --- | --- | --- | --- | --- |
| Week 0 | BioPatty vs. Vehicle Patty | 0.000 |  |  |
|  | BioPatty vs. BioSpray | -0.316 | -1.420 to 0.788 | 0.838 |
|  | BioPatty vs. Vehicle Spray | -0.156 | -0.460 to 0.149 | 0.451 |
|  | BioPatty vs. NTC | -1.027 | -2.722 to 0.667 | 0.291 |
|  | Vehicle Patty vs. BioSpray | -0.316 | -1.420 to 0.788 | 0.838 |
|  | Vehicle Patty vs. Vehicle Spray | -0.156 | -0.460 to 0.149 | 0.451 |
|  | Vehicle Patty vs. NTC | -1.027 | -2.722 to 0.667 | 0.291 |
|  | BioSpray vs. Vehicle Spray | 0.160 | -0.944 to 1.264 | 0.985 |
|  | BioSpray vs. NTC | -0.712 | -2.512 to 1.089 | 0.719 |
|  | Vehicle Spray vs. NTC | -0.872 | -2.564 to 0.821 | 0.433 |
| Week 4 | BioPatty vs. Vehicle Patty | 0.331 | -4.893 to 5.556 | 0.999 |
|  | BioPatty vs. BioSpray | -0.180 | -5.363 to 5.003 | 0.999 |
|  | BioPatty vs. Vehicle Spray | 0.151 | -5.045 to 5.347 | 0.999 |
|  | BioPatty vs. NTC | 1.156 | -4.640 to 6.952 | 0.844 |
|  | Vehicle Patty vs. BioSpray | -0.511 | -4.049 to 3.027 | 0.990 |
|  | Vehicle Patty vs. Vehicle Spray | -0.180 | -4.002 to 3.642 | 1.000 |
|  | Vehicle Patty vs. NTC | 0.825 | -2.356 to 4.006 | 0.876 |
|  | BioSpray vs. Vehicle Spray | 0.331 | -3.173 to 3.835 | 0.998 |
|  | BioSpray vs. NTC | 1.336 | -1.227 to 3.900 | 0.443 |
|  | Vehicle Spray vs. NTC | 1.005 | -2.080 to 4.090 | 0.790 |
| Week 8 | BioPatty vs. Vehicle Patty | -0.040 | -5.418 to 5.337 | 0.999 |
|  | BioPatty vs. BioSpray | -0.408 | -5.350 to 4.534 | 0.998 |
|  | BioPatty vs. Vehicle Spray | -1.839 | -6.809 to 3.130 | 0.634 |
|  | BioPatty vs. NTC | -1.920 | -6.954 to 3.114 | 0.702 |
|  | Vehicle Patty vs. BioSpray | -0.368 | -4.501 to 3.765 | 0.998 |
|  | Vehicle Patty vs. Vehicle Spray | -1.799 | -5.860 to 2.263 | 0.504 |
|  | Vehicle Patty vs. NTC | -1.880 | -6.174 to 2.414 | 0.616 |
|  | BioSpray vs. Vehicle Spray | -1.431 | -3.858 to 0.9966 | 0.348 |
|  | BioSpray vs. NTC | -1.512 | -4.821 to 1.797 | 0.609 |
|  | Vehicle Spray vs. NTC | -0.081 | -3.042 to 2.879 | 0.999 |

**Table S11.** Two-way analysis of variance (ANOVA) and Tukey’s multiple comparisons test of *P. larvae* loads for all five treatment groups (n=3 hives per group) at each week (n=3 timepoints) for the agricultural location. Tukey HSD tests results are bolded for significant difference between treatment groups within a single timepoint.

|  | **Tukey's multiple comparisons test** | **Mean Diff.** | **95.00% CI of diff.** | **Adjusted *P* value** |
| --- | --- | --- | --- | --- |
| Week 0 | BioPatty vs. Vehicle Patty | -0.065 | -0.378 to 0.248 | 0.946 |
|  | BioPatty vs. BioSpray | 0.022 | -0.059 to 0.103 | 0.853 |
|  | BioPatty vs. Vehicle Spray | -0.007 | -0.123 to 0.109 | 0.999 |
|  | BioPatty vs. NTC | 0.008 | -0.077 to 0.093 | 0.998 |
|  | Vehicle Patty vs. BioSpray | 0.087 | -0.226 to 0.400 | 0.849 |
|  | Vehicle Patty vs. Vehicle Spray | 0.058 | -0.256 to 0.372 | 0.967 |
|  | Vehicle Patty vs. NTC | 0.073 | -0.239 to 0.386 | 0.914 |
|  | BioSpray vs. Vehicle Spray | -0.029 | -0.132 to 0.073 | 0.853 |
|  | BioSpray vs. NTC | -0.014 | -0.063 to 0.035 | 0.857 |
|  | Vehicle Spray vs. NTC | 0.016 | -0.090 to 0.121 | 0.989 |
| Week 4 | BioPatty vs. Vehicle Patty | -0.244 | -1.030 to 0.542 | 0.862 |
|  | BioPatty vs. BioSpray | 0.146 | -0.332 to 0.625 | 0.805 |
|  | BioPatty vs. Vehicle Spray | 0.152 | -0.326 to 0.630 | 0.785 |
|  | BioPatty vs. NTC | -0.269 | -1.152 to 0.613 | 0.864 |
|  | Vehicle Patty vs. BioSpray | 0.390 | -0.341 to 1.121 | 0.414 |
|  | Vehicle Patty vs. Vehicle Spray | 0.396 | -0.336 to 1.127 | 0.402 |
|  | Vehicle Patty vs. NTC | -0.026 | -1.016 to 0.965 | 0.999 |
|  | BioSpray vs. Vehicle Spray | 0.005 | -0.014 to 0.025 | 0.848 |
|  | BioSpray vs. NTC | -0.416 | -1.257 to 0.426 | 0.480 |
|  | Vehicle Spray vs. NTC | -0.421 | -1.263 to 0.420 | 0.469 |
| Week 8 | BioPatty vs. Vehicle Patty | -0.300 | -1.375 to 0.774 | 0.848 |
|  | BioPatty vs. BioSpray | 0.000 |  |  |
|  | BioPatty vs. Vehicle Spray | -0.108 | -0.419 to 0.203 | 0.752 |
|  | BioPatty vs. NTC | -0.157 | -0.700 to 0.385 | 0.831 |
|  | Vehicle Patty vs. BioSpray | 0.300 | -0.774 to 1.375 | 0.848 |
|  | Vehicle Patty vs. Vehicle Spray | 0.192 | -0.883 to 1.267 | 0.969 |
|  | Vehicle Patty vs. NTC | 0.143 | -0.9567to 1.243 | 0.992 |
|  | BioSpray vs. Vehicle Spray | -0.108 | -0.419 to 0.203 | 0.752 |
|  | BioSpray vs. NTC | -0.157 | -0.700 to 0.385 | 0.831 |
|  | Vehicle Spray vs. NTC | -0.049 | -0.615 to 0.516 | 0.999 |

| **Table S12.** Tukey’s multiple comparisons test of bacterial (BAC) and *P. larvae* loads at week 0 for all locations (agricultural, forage-rich, and urban).   \| **Week 0** \| **Tukey's multiple**  **comparisons test** \| **Mean Diff.** \| **95.00% CI of diff.** \| **Summary** \| **Adjust *P* Value** \| \| --- \| --- \| --- \| --- \| --- \| --- \| \| BAC \| BioPatty vs. Vehicle Patty \| 0.007 \| -0.759 to 0.773 \| ns \| 0.999 \| \| BioPatty vs. BioSpray \| -0.260 \| -0.997 to 0.478 \| ns \| 0.869 \| \| BioPatty vs. Vehicle Spray \| 0.035 \| -0.688 to 0.757 \| ns \| 0.999 \| \| BioPatty vs. NTC \| -0.460 \| -1.190 to 0.270 \| ns \| 0.415 \| \| Vehicle Patty vs. BioSpray \| -0.266 \| -1.033 to 0.500 \| ns \| 0.874 \| \| Vehicle Patty vs. Vehicle Spray \| 0.028 \| -0.724 to 0.779 \| ns \| 0.999 \| \| Vehicle Patty vs. NTC \| -0.467 \| -1.225 to 0.292 \| ns \| 0.441 \| \| BioSpray vs. Vehicle Spray \| 0.294 \| -0.428 to 1.016 \| ns \| 0.796 \| \| BioSpray vs. NTC \| -0.200 \| -0.930 to 0.529 \| ns \| 0.943 \| \| Vehicle Spray vs. NTC \| -0.494 \| -1.208 to 0.219 \| ns \| 0.318 \| \| *P. larvae* \| BioPatty vs. Vehicle Patty \| -0.129 \| -0.895 to 0.638 \| ns \| 0.991 \| \| BioPatty vs. BioSpray \| -0.020 \| -0.758 to 0.718 \| ns \| 0.999 \| \| BioPatty vs. Vehicle Spray \| 0.026 \| -0.696 to 0.748 \| ns \| 0.999 \| \| BioPatty vs. NTC \| -0.136 \| -0.866 to 0.593 \| ns \| 0.986 \| \| Vehicle Patty vs. BioSpray \| 0.109 \| -0.658 to 0.875 \| ns \| 0.995 \| \| Vehicle Patty vs. Vehicle Spray \| 0.155 \| -0.597 to 0.906 \| ns \| 0.980 \| \| Vehicle Patty vs. NTC \| -0.008 \| -0.766 to 0.751 \| ns \| 0.999 \| \| BioSpray vs. Vehicle Spray \| 0.046 \| -0.676 to 0.768 \| ns \| 1.000 \| \| BioSpray vs. NTC \| -0.116 \| -0.846 to 0.614 \| ns \| 0.992 \| \| Vehicle Spray vs. NTC \| -0.162 \| -0.876 to 0.552 \| ns \| 0.971 \|   **Table S13.** Tukey’s Multiple Comparisons test of bacterial (BAC) and *P. larvae* loads at week 4 for all locations (agricultural, forage-rich, and urban).   \| **Week 4** \| **Tukey's multiple**  **comparisons test** \| **Mean Diff.** \| **95.00% CI of diff.** \| **Summary** \| **Adjust *P* Value** \| \| --- \| --- \| --- \| --- \| --- \| --- \| \| BAC \| BioPatty vs. Vehicle Patty \| 0.029 \| -0.987 to 1.044 \| ns \| 0.999 \| \| BioPatty vs. BioSpray \| -0.040 \| -1.009 to 0.929 \| ns \| 0.999 \| \| BioPatty vs. Vehicle Spray \| 0.351 \| -0.609 to 1.311 \| ns \| 0.852 \| \| BioPatty vs. NTC \| -0.356 \| -1.326 to 0.613 \| ns \| 0.850 \| \| Vehicle Patty vs. BioSpray \| -0.069 \| -1.051 to 0.914 \| ns \| 1.000 \| \| Vehicle Patty vs. Vehicle Spray \| 0.322 \| -0.651 to 1.296 \| ns \| 0.892 \| \| Vehicle Patty vs. NTC \| -0.385 \| -1.368 to 0.597 \| ns \| 0.817 \| \| BioSpray vs. Vehicle Spray \| 0.391 \| -0.534 to 1.316 \| ns \| 0.772 \| \| BioSpray vs. NTC \| -0.317 \| -1.251 to 0.618 \| ns \| 0.884 \| \| Vehicle Spray vs. NTC \| -0.708 \| -1.633 to 0.217 \| ns \| 0.222 \| \| *P. larvae* \| BioPatty vs. Vehicle Patty \| -0.259 \| -1.274 to 0.757 \| ns \| 0.956 \| \| BioPatty vs. BioSpray \| -0.178 \| -1.147 to 0.792 \| ns \| 0.987 \| \| BioPatty vs. Vehicle Spray \| -0.070 \| -1.030 to 0.890 \| ns \| 1.000 \| \| BioPatty vs. NTC \| 0.111 \| -0.858 to 1.080 \| ns \| 0.998 \| \| Vehicle Patty vs. BioSpray \| 0.081 \| -0.901 to 1.064 \| ns \| 0.999 \| \| Vehicle Patty vs. Vehicle Spray \| 0.189 \| -0.784 to 1.162 \| ns \| 0.984 \| \| Vehicle Patty vs. NTC \| 0.370 \| -0.613 to 1.353 \| ns \| 0.839 \| \| BioSpray vs. Vehicle Spray \| 0.108 \| -0.817 to 1.033 \| ns \| 0.998 \| \| BioSpray vs. NTC \| 0.289 \| -0.646 to 1.223 \| ns \| 0.915 \| \| Vehicle Spray vs. NTC \| 0.181 \| -0.744 to 1.106 \| ns \| 0.983 \|   **Table S14.** Tukey’s Multiple Comparisons test of bacterial (BAC) and *P. larvae* loads at week 8 for all locations (agricultural, forage-rich, and urban).   \| **Week 8** \| **Tukey's multiple**  **comparisons test** \| **Mean Diff.** \| **95.00% CI of diff.** \| **Summary** \| **Adjust *P* Value** \| \| --- \| --- \| --- \| --- \| --- \| --- \| \| BAC \| BioPatty vs. Vehicle Patty \| -0.022 \| -1.065 to 1.020 \| ns \| 0.999 \| \| BioPatty vs. BioSpray \| -0.124 \| -1.119 to 0.871 \| ns \| 0.997 \| \| BioPatty vs. Vehicle Spray \| -0.269 \| -1.264 to 0.726 \| ns \| 0.946 \| \| BioPatty vs. NTC \| -0.751 \| -1.756 to 0.255 \| ns \| 0.244 \| \| Vehicle Patty vs. BioSpray \| -0.102 \| -1.134 to 0.931 \| ns \| 0.999 \| \| Vehicle Patty vs. Vehicle Spray \| -0.247 \| -1.279 to 0.785 \| ns \| 0.965 \| \| Vehicle Patty vs. NTC \| -0.728 \| -1.771 to 0.314 \| ns \| 0.309 \| \| BioSpray vs. Vehicle Spray \| -0.145 \| -1.130 to 0.839 \| ns \| 0.994 \| \| BioSpray vs. NTC \| -0.627 \| -1.621 to 0.368 \| ns \| 0.416 \| \| Vehicle Spray vs. NTC \| -0.481 \| -1.476 to 0.514 \| ns \| 0.672 \| \| *P. larvae* \| BioPatty vs. Vehicle Patty \| 0.339 \| -0.718 to 1.396 \| ns \| 0.903 \| \| BioPatty vs. BioSpray \| 0.387 \| -0.608 to 1.382 \| ns \| 0.822 \| \| BioPatty vsVehicle Spray \| 0.256 \| -0.739 to 1.250 \| ns \| 0.955 \| \| BioPatty vs. NTC \| 0.256 \| -0.750 to 1.261 \| ns \| 0.956 \| \| Vehicle Patty vs. BioSpray \| 0.048 \| -0.999 to 1.095 \| ns \| 0.999 \| \| Vehicle Patty vs. Vehicle Spray \| -0.084 \| -1.131 to 0.963 \| ns \| 1.000 \| \| Vehicle Patty vs. NTC \| -0.083 \| -1.140 to 0.974 \| ns \| 1.000 \| \| BioSpray vs. Vehicle Spray \| -0.131 \| -1.115 to 0.853 \| ns \| 0.996 \| \| BioSpray vs. NTC \| -0.131 \| -1.126 to 0.864 \| ns \| 0.996 \| \| Vehicle Spray vs. NTC \| 0.000 \| -0.995 to 0.995 \| ns \| 0.999 \|   **Table S15.** Mixed-Effects Analysis of bacterial (BAC) loads for all locations and treatment groups. | | | | | |
| --- | --- | --- | --- | --- | --- | --- | --- | --- | --- | --- | --- | --- | --- | --- | --- | --- | --- | --- | --- | --- | --- | --- | --- | --- | --- | --- | --- | --- | --- | --- | --- | --- | --- | --- | --- | --- | --- | --- | --- | --- | --- | --- | --- | --- | --- | --- | --- | --- | --- | --- | --- | --- | --- | --- | --- | --- | --- | --- | --- | --- | --- | --- | --- | --- | --- | --- | --- | --- | --- | --- | --- | --- | --- | --- | --- | --- | --- | --- | --- | --- | --- | --- | --- | --- | --- | --- | --- | --- | --- | --- | --- | --- | --- | --- | --- | --- | --- | --- | --- | --- | --- | --- | --- | --- | --- | --- | --- | --- | --- | --- | --- | --- | --- | --- | --- | --- | --- | --- | --- | --- | --- | --- | --- | --- | --- | --- | --- | --- | --- | --- | --- | --- | --- | --- | --- | --- | --- | --- | --- | --- | --- | --- | --- | --- | --- | --- | --- | --- | --- | --- | --- | --- | --- | --- | --- | --- | --- | --- | --- | --- | --- | --- | --- | --- | --- | --- | --- | --- | --- | --- | --- | --- | --- | --- | --- | --- | --- | --- | --- | --- | --- | --- | --- | --- | --- | --- | --- | --- | --- | --- | --- | --- | --- | --- | --- | --- | --- | --- | --- | --- | --- | --- | --- | --- | --- | --- | --- | --- | --- | --- | --- | --- | --- | --- | --- | --- | --- | --- | --- | --- | --- | --- | --- | --- | --- | --- | --- | --- | --- | --- | --- | --- | --- | --- | --- | --- | --- | --- | --- | --- | --- | --- | --- | --- | --- | --- | --- | --- | --- | --- | --- | --- | --- | --- | --- | --- | --- | --- | --- | --- | --- | --- | --- | --- | --- | --- | --- | --- | --- | --- | --- | --- | --- | --- | --- | --- | --- | --- | --- | --- | --- | --- | --- | --- | --- | --- | --- | --- | --- | --- | --- | --- | --- | --- | --- | --- | --- | --- | --- | --- | --- | --- | --- | --- | --- | --- | --- | --- | --- | --- | --- | --- | --- | --- | --- | --- | --- | --- | --- | --- | --- | --- | --- | --- | --- | --- | --- | --- | --- |
| **Fixed effects**  **(type III)** | ***P* value** | ***P* value summary** | **Statistically significant (P < 0.05)?** | **F _(DFn, DFd)_** | **Geisser-Greenhouse's epsilon** |
| Week | 0.0007 | *** | Yes | F _(1.601, 140.1)_ = 8.901 | 0.8005 |
| Treatment | 0.4686 | ns | No | F _(4, 116)_ = 0.8964 |  |
| Week x Treatment | 0.8895 | ns | No | F _(8, 175)_ = 0.4497 |  |

**Table S16.** Tukey’s Multiple Comparisons test of bacterial (BAC) loads for all locations (n=3 sites) and treatment groups (n=5 groups).

|  | **Tukey's multiple comparisons test** | **Mean Diff.** | **95% CI of diff.** | **Adjusted *P* Value** |
| --- | --- | --- | --- | --- |
| BioPatty | Week 0 vs. Week 4 | 0.144 | -0.116 to 0.405 | 0.358 |
|  | Week 0 vs. Week 8 | -0.282 | -0.982 to 0.418 | 0.567 |
|  | Week 4 vs. Week 8 | -0.426 | -1.161 to 0.309 | 0.322 |
|  |  |  |  |  |
| Vehicle Patty | Week 0 vs. Week 4 | -0.089 | -0.591 to 0.412 | 0.358 |
|  | Week 0 vs. Week 8 | -0.372 | -0.962 to 0.219 | 0.567 |
|  | Week 4 vs. Week 8 | -0.282 | -0.953 to 0.389 | 0.322 |
|  |  |  |  |  |
| BioSpray | Week 0 vs. Week 4 | -0.177 | -0.461 to 0.106 | 0.274 |
|  | Week 0 vs. Week 8 | -0.296 | -0.718 to 0.127 | 0.202 |
|  | Week 4 vs. Week 8 | -0.118 | -0.448 to 0.212 | 0.639 |
|  |  |  |  |  |
| Vehicle Spray | Week 0 vs. Week 4 | -0.085 | -0.475 to 0.304 | 0.840 |
|  | Week 0 vs. Week 8 | -0.257 | -0.877 to 0.363 | 0.539 |
|  | Week 4 vs. Week 8 | -0.172 | -0.945 to 0.602 | 0.835 |
|  |  |  |  |  |
| NTC | Week 0 vs. Week 4 | -0.168 | -0.522 to 0.186 | 0.468 |
|  | Week 0 vs. Week 8 | -0.540 | -0.923 to -0.157 | **0.005** |
|  | Week 4 vs. Week 8 | -0.372 | -0.803 to 0.059 | 0.098 |

| **Table S17.** Mixed-Effects Analysis of *P. larvae* loads for all locations (n=3 sites) and treatment groups (n=5 groups). | | | |
| --- | --- | --- | --- |
| **Fixed effects(type III)** | ***P* value** | **F _(DFn, DFd)_** | **Geisser-Greenhouse's epsilon** |
| Week | 0.0925 | F _(1.730, 302.8)_ = 2.491 | 0.8651 |
| Treatment | 0.9466 | F _(4, 350)_ = 0.1841 |  |
| Week x Treatment | 0.6035 | F _(8, 350)_ = 0.7993 |  |

**Table S18.** Tukey’s multiple comparisons test of *P. larvae* loads for all locations (n=3 sites) and treatment groups (n=5 groups).

|  | **Tukey's multiple comparisons test** | **Mean Diff.** | **95% CI of diff.** | **Adjusted *P* Value** |
| --- | --- | --- | --- | --- |
| BioPatty | Week 0 vs. Week 4 | -0.218 | -0.854 to 0.418 | 0.666 |
|  | Week 0 vs. Week 8 | -0.232 | -0.959 to 0.495 | 0.704 |
|  | Week 4 vs. Week 8 | -0.014 | -0.941 to 0.913 | 0.999 |
|  |  |  |  |  |
| Vehicle Patty | Week 0 vs. Week 4 | -0.305 | -0.775 to 0.164 | 0.249 |
|  | Week 0 vs. Week 8 | 0.081 | -0.310 to 0.472 | 0.860 |
|  | Week 4 vs. Week 8 | 0.386 | -0.317 to 1.090 | 0.367 |
|  |  |  |  |  |
| BioSpray | Week 0 vs. Week 4 | -0.489 | -1.236 to 0.259 | 0.250 |
|  | Week 0 vs. Week 8 | 0.105 | -0.147 to 0.357 | 0.558 |
|  | Week 4 vs. Week 8 | 0.594 | -0.067 to 1.255 | 0.084 |
|  |  |  |  |  |
| Vehicle Spray | Week 0 vs. Week 4 | -0.331 | -1.165 to 0.504 | 0.584 |
|  | Week 0 vs. Week 8 | -0.046 | -0.316 to 0.223 | 0.903 |
|  | Week 4 vs. Week 8 | 0.284 | -0.522 to 1.09 | 0.656 |
|  |  |  |  |  |
| NTC | Week 0 vs. Week 4 | 0.139 | -0.204 to 0.482 | 0.571 |
|  | Week 0 vs. Week 8 | -0.010 | -0.854 to 0.834 | 1.000 |
|  | Week 4 vs. Week 8 | -0.149 | -0.543 to 0.244 | 0.613 |
